# Effects of Myofibrillar Protein-Stabilized Soybean Oil Pre-Emulsions Containing Arabic Gum or Gallic Acid-Grafted Arabic Gum on the Quality Characteristics and Oxidative Stability of Reduced-Animal-Fat Emulsified Sausages

**DOI:** 10.3390/foods15162795

**Published:** 2026-08-10

**Authors:** Chang Liu, Mingze Xu, Yi Luan, Khubaib Ali, Rui Sun, Qingling Wang, Shixue Wu, Yixue Shi, Mangang Wu

**Affiliations:** College of Food Science and Engineering, Yangzhou University, Yangzhou 225127, China; 19825303901@163.com (C.L.); xumingze45@gmail.com (M.X.); ly19980000@163.com (Y.L.); khubaibalee83@gmail.com (K.A.); sui97599759@163.com (R.S.); 18133432613@139.com (S.W.); nineplanetex@163.com (Y.S.)

**Keywords:** gallic acid-grafted Arabic gum, soybean oil pre-emulsion, lipid oxidation, oxidative stability

## Abstract

Soybean oil-based pre-emulsions stabilized by myofibrillar protein (MP), Arabic gum (AG), or gallic acid-grafted Arabic gum (GA-AG) were used to partially (50%) or fully (100%) replace pork back fat in Western-style emulsified sausages. The present study investigated the effects of such replacement on the initial quality attributes and lipid oxidation stability of sausages during storage. Compared with sausages formulated with pure pork back fat, pre-emulsion substitution significantly reduced cooking loss and improved textural characteristics, especially hardness and springiness. Incorporating AG into the soybean oil pre-emulsion further enhanced the cooking yield of the products, while GA-AG retained AG’s stabilizing efficacy without compromising sensory acceptability. Sensory evaluation results demonstrated that 50% fat replacement achieved an optimal balance between textural performance and overall sensory preference. During refrigerated storage, the thiobarbituric acid reactive substance (TBARS) values increased in all sausage formulations. In contrast, sausages formulated with the GA-AG-stabilized soybean oil pre-emulsion exhibited the most potent inhibitory effect on lipid oxidation. After 312 h of storage, the TBARS values of the GA-AG groups were merely 32.8% of those of the single MP-treated groups. These findings suggest that the MP/GA-AG-stabilized soybean oil pre-emulsion system effectively improves the processing properties and oxidative stability of low-animal-fat emulsified sausages.

## 1. Introduction

Emulsified meat products, such as frankfurter-type sausages, are an important category of processed meat and are widely consumed owing to their nutritional value and desirable sensory attributes. They provide high-quality protein, essential amino acids, vitamins, and minerals, and offer characteristic flavor, texture, and convenience [1]. In conventional formulations, animal fat is an essential ingredient that contributes to juiciness, tenderness, mouthfeel, and flavor release, as well as to the formation of a stable meat emulsion-gel matrix [2]. However, emulsified meat products generally contain high levels of fat, approximately 20–35 g/100 g, and excessive intake of high-fat processed meat products has raised nutritional concerns due to increased energy intake and dietary fat consumption [3]. The composition and nutritional characteristics of animal fat can vary depending on factors such as animal breed, feeding conditions, and anatomical location. Therefore, current strategies for reformulating emulsified meat products mainly focus on reducing animal fat content while maintaining desirable technological and sensory properties to improve overall product quality [4]. 

To address these technological and quality-related challenges, various fat-reduction and fat-modification strategies have been proposed for developing healthier emulsified meat products. Among them, the incorporation of pre-emulsified oils as animal fat replacers has attracted considerable attention [5]. This strategy can alleviate the quality defects associated with animal fat reduction, including oil and water exudation, impaired emulsion stability, and deterioration of sensory attributes [6]. Moreover, replacing animal fat with pre-emulsified vegetable oils represents a promising strategy for reducing animal fat incorporation and tailoring the lipid characteristics of emulsified sausages. However, the functional and nutritional implications of such substitution may vary depending on the characteristics of the selected oils and the final product formulation. Nevertheless, pre-emulsified oil systems are thermodynamically unstable and require suitable emulsifying stabilizers to maintain droplet dispersion and prevent phase separation [7]. Moreover, such systems are more susceptible to oxidative deterioration owing to the high degree of unsaturation in vegetable oils, which may cause lipid oxidation products to accumulate during processing and storage, thereby compromising the flavor, color, texture, and overall quality of the products [8]. In emulsified meat systems, myofibrillar protein (MP) is the major structure-forming protein, responsible for interfacial adsorption, fat immobilization, and heat-induced gel network formation [9]. During emulsification, MP can partially unfold and adsorb around oil droplets to form an interfacial film, and the emulsified droplets are subsequently embedded within the protein gel network after heating. These structural features are critical for water and oil retention, textural stability, and processing performance in emulsified meat products [10]. However, MP-stabilized oil droplets alone provide limited long-term protection against lipid oxidation during refrigerated storage, particularly when vegetable oils rich in unsaturated fatty acids are used as animal fat replacers [7]. Therefore, constructing a composite interfacial layer that combines physical stabilization with antioxidant functionality may be an effective strategy for improving the stability of pre-emulsified vegetable oil systems.

Naturally derived polysaccharides have been widely explored as emulsifying stabilizers, owing to their ability to increase aqueous-phase viscosity and to provide steric hindrance and interfacial stability [11]. Arabic gum (AG) is a natural branched polysaccharide composed mainly of arabinogalactan and glycoprotein fractions. Owing to its high-water solubility and interfacial adsorption capacity, AG has been widely used as an emulsifier and stabilizer in the food industry. However, native AG provides mainly physical stabilization and exhibits limited antioxidant activity, which restricts its effectiveness in lipid-rich food systems, where oxidative deterioration during storage is a major cause of quality loss and shelf-life reduction. To overcome this limitation, structural modification of polysaccharides has attracted increasing attention as an effective strategy for improving their functional and antioxidant properties [12]. In particular, the covalent grafting of phenolic acids onto polysaccharide backbones has emerged as an important approach to enhancing antioxidant capacity [13]. This modification can improve radical-scavenging and metal-chelating abilities while preserving interfacial adsorption behavior. Our previous work demonstrated that grafting phenolic acids onto polysaccharides effectively improved the oxidative stability of the resulting emulsions and emulsion gels. Among three representative phenolic acids, namely caffeic acid, gallic acid (GA), and ferulic acid, GA, a trihydroxybenzoic acid, conferred the most pronounced ability to inhibit lipid oxidation and enhance oxidative stability in emulsion and emulsion gel systems. However, these findings were obtained mainly from model emulsion systems [14]. Whether GA-modified AG (GA-AG) can cooperate with MP to stabilize vegetable oil droplets and provide interfacial antioxidant protection in a real emulsified meat matrix remains unclear.

To address this knowledge gap, this study constructed an MP and GA-AG -stabilized vegetable oil pre-emulsion based on the previously developed GA-AG system and applied it in Western-style emulsified sausages for partial (50%) and complete (100%) replacement of animal fat. The effects of the composite pre-emulsion on the physicochemical properties, cooking loss, textural characteristics, sensory quality, and lipid oxidation stability of the sausages were systematically evaluated. This study was expected to clarify the practical functionality of the MP and GA-AG composite system in a real meat batter matrix and to provide a multifunctional strategy for enhancing emulsion stability, controlling lipid oxidation, and improving the quality preservation of reduced-animal-fat emulsified meat products during storage.

## 2. Materials and Methods

### 2.1. Materials and Reagents

Pork longissimus dorsi, pork back fat, and soybean oil were purchased from Auchan Supermarket (Yangzhou, China). The pork longissimus dorsi muscle and pork back fat were obtained from the same commercial source and selected under consistent conditions. Before processing, visible connective tissues and external fats were carefully removed from the lean meat. The pork longissimus dorsi muscle was used as the primary lean meat source for myofibrillar protein extraction and sausage matrix formation. In contrast, pork back fat served as the conventional animal fat source in the control formulation. Ethylene glycol-bis(β-aminoethyl ether)-N,N,N′,N′-tetraacetic acid (EGTA, CAS#67-42-5), ethylenediaminetetraacetic acid (EDTA, CAS#60-00-4), and trichloroacetic acid (CAS#76-03-9) were purchased from Sinopharm Chemical Reagent Co., Ltd. (Shanghai, China). Thiobarbituric acid (TBA, CAS#504-17-6) was obtained from Aladdin Reagent Co., Ltd. (Shanghai, China). AG (CAS#9000-01-5) and laccase (15 LAMU/g, CAS#80498-15-3) were supplied by Sangon Biotech Co., Ltd. (Shanghai, China). GA (≥99% purity, CAS#149-91-7) was purchased from Shanghai Yuanye Bio-Technology Co., Ltd. (Shanghai, China). All other chemicals and reagents were of analytical grade.

### 2.2. Preparation of GA-AG

GA-AG was prepared according to the methods of Karaki et al. [15] and Vuillemin et al. [12], with slight modifications. Briefly, AG (1.0 g) was dispersed in phosphate buffer (45 mL, 0.2 mol/L, pH 7.5) and stirred until complete dissolution. A methanolic GA solution (5 mL, 50 mM) and laccase (15 LAMU/g) were then added sequentially to the AG solution. The reaction mixture was stirred at 1000 rpm for 4 h under atmospheric conditions without limiting dissolved oxygen. The reaction was terminated by adding an equal volume of ethanol, followed by centrifugation at 6000× *g* for 20 min to remove water-insoluble fractions. Ethanol and part of the water were removed from the supernatant using a rotary evaporator at 40–50 °C. The concentrate obtained by rotary evaporation was dialyzed against ultrapure water using a biotech-grade dialysis membrane (MWCO 10,000 Da; Labest Biotechnology Co., Ltd., Beijing, China) to remove residual salts, free GA, and other low-molecular-weight compounds. The purified solution was freeze-dried for 72 h, and the obtained GA-AG powder was sealed and stored in a dry environment until further use.

### 2.3. Extraction of MP

MPs were extracted according to the method of Park et al. [16]. The extraction process was always operated at 4 °C. Pre-cut porcine longissimus muscle was thawed at 4 °C, and visible connective tissues were removed. The muscle was then cut along the fiber orientation and homogenized using a tissue grinder. Phosphate buffer (10 mM Na_2_HPO_4_·12H_2_O, 0.1 M NaCl, 2 mM MgCl_2_·6H_2_O, 1 mM EGTA, pH 7.0) at four times the muscle volume was added, and the mixture was homogenized at a constant speed for 1 min. The resulting homogenate was centrifuged at 2000× *g* for 15 min, and the supernatant was discarded. This centrifugation step was repeated three times. The pellet was resuspended in 0.1 M NaCl (4× volume), homogenized, and filtered to remove residual connective tissue. After a final centrifugation, the resulting pellet was collected as MP, stored at 4 °C, and used within 48 h. The protein concentration was determined using the Biuret method, with BSA as the standard protein [17].

### 2.4. Preparation of Compound Emulsions

Plant oil pre-emulsions were prepared according to the method of Yu and Zhang [18], with slight modifications. Three types of aqueous phases were prepared for the different pre-emulsion systems. For the MP-stabilized pre-emulsion, MP was dissolved in phosphate buffer to obtain an aqueous phase containing 1.0% MP. For the MP-AG and MP-GA-AG composite pre-emulsions, an MP solution (2.0%, *w*/*w*) was mixed with AG or GA-AG solution (1.2%, *w*/*w*) at a 1:1 ratio, followed by hydration to obtain composite solutions containing 1.0% MP and 0.6% AG or GA-AG. Soybean oil was subsequently combined with each corresponding aqueous phase at an oil-to-aqueous phase mass ratio of 20:80, such that soybean oil accounted for 20% (*w*/*w*) of the total pre-emulsion mass, followed by homogenization using a high-speed homogenizer (ULTRA-TURRAX T18 digital, IKA, Staufen, Germany) at 10,000 rpm for 1 min. The resulting emulsions were designated as MP, MP-AG, and MP-MAG pre-emulsions, respectively, where MAG denotes modified Arabic gum prepared through gallic acid grafting (GA-AG). All procedures were carried out at 4 ℃. 

### 2.5. Preparation of Western-Style Emulsified Sausage

#### 2.5.1. Raw Material Processing and Formulation Design

Western-style emulsified sausages were prepared according to the method described by Zhong et al. [19] with slight modifications. Fresh pork longissimus dorsi and pork back fat were used as raw materials. Visible fat and connective tissues were carefully removed. Both the lean meat and back fat were separately comminuted using a meat grinder. To ensure statistical rigor, each sausage formulation was processed individually from raw material weighing to final storage, with each batch serving as an independent experimental unit rather than a subsample. The specific formulations are detailed in Table 1. The control group, prepared exclusively with pork back fat, was designated as CK. For the fat-reduction treatments, the nominal replacement level was defined as the proportion of the 30 g of pork back fat in the control formulation that was removed. Specifically, the 50% and 100% replacement formulations contained 15 and 0 g of pork back fat and incorporated 30.4 and 60.4 g of the corresponding pre-emulsion, respectively, thereby providing 6.08 and 12.08 g of soybean oil, while the amount of ice was adjusted to maintain a constant total batch mass. Therefore, the replacement levels refer to the proportion of pork back fat removed rather than to an equivalent replacement of lipid. Based on the stabilizer type and replacement level, the experimental groups were denoted as MP-50, MP-100, MP-AG-50, MP-AG-100, MP-GA-AG-50, and MP-GA-AG-100, respectively.

#### 2.5.2. Chopping Procedures

The chopping process was conducted independently for each batch. The addition sequence and volume of ice water were systematically adjusted according to the fat replacement strategy to optimize emulsion stability. Throughout the chopping process, the core temperature of the meat batter was strictly maintained below 10 °C to prevent premature protein denaturation. The specific chopping sequences for the different treatment groups were executed as follows:

The CK group: The lean meat was initially homogenized with salt, phosphate, and one-third of the total ice water for 1 min. Following a 2-min rest, the pork back fat and another one-third of the ice water were added, followed by chopping for 1 min. Finally, the remaining ice water was incorporated, and the mixture was chopped for an additional 3 min to form a uniform meat batter.

The 50% replacement groups: The lean meat was first chopped with salt, phosphate, and half of the total ice water for 1 min. After a 2-min rest, the respective plant oil pre-emulsion was introduced and chopped for 1 min. After another 2-min pause, the pork back fat and the remaining ice water were added, followed by a final 3-min chopping cycle.

The 100% replacement groups: The lean meat was initially comminuted with salt, phosphate, and the entire volume of ice water for 1 min. After a 2-min rest, the corresponding plant oil pre-emulsion was fully incorporated, and the batter was chopped continuously for 3 min to achieve a homogeneous matrix.

#### 2.5.3. Thermal Processing

After chopping, each independent meat batter was immediately stuffed into polyethylene casings (20 mm in diameter). The stuffed sausages were thermally processed in a water bath at 80 °C for 30 min, subsequently chilled in ice water, vacuum-packaged, and stored at 4 °C for further analysis.

### 2.6. Emulsified Sausage Color and pH Value Determination

The color of emulsified sausages was measured using a colorimeter (CM-700d, Konica Minolta, Sakai, Osaka, Japan). The instrument was calibrated with a standard white calibration plate before measurement. Color measurements were performed using the CIE Lab color system under a D65 illuminant with a 10° standard observer angle. The measurement aperture was set to MAV (8 mm), and the instrument was operated according to the manufacturer’s instructions. For each formulation, independently prepared sausage samples from each batch were used for color evaluation. Color measurements were performed at the initial stage (0 h) after sample preparation. Samples stored at 4 °C were equilibrated to room temperature (approximately 25 °C) before measurement and cut to expose a fresh cross-sectional surface. The cut surfaces were measured immediately after exposure without additional blooming time. The L*, a*, and b* values were measured at three randomly selected positions on the cross section of each sample, and the mean value was recorded.

(1) $$C^{*}={\left(a^{*2}+b^{*2}\right)}^{\frac{1}{2}}$$

where L* is the brightness value, a* is the redness value, and b* is the yellowness value. Chromaticity (C*) is calculated according to the following formula [20].

The pH of emulsified sausages was measured at the initial stage (0 h) using a portable penetration pH meter (Testo 205, Testo SE & Co. KGaA, Lenzkirch, Germany). The instrument was calibrated with standard buffer solutions before measurement. Three measurements were performed for each sample, and the mean value was recorded.

### 2.7. Determination of Cooking Loss of Emulsified Sausages

Cooking loss was determined by weighing the emulsified sausages before and after thermal processing. Briefly, the stuffed sausages were weighed before heating, cooled to room temperature after cooking, gently wiped to remove surface moisture, and weighed again. Cooking loss was calculated according to the following equation.

(2) $$\mathrm{Cooking}\;\mathrm{loss}\;\left(\%\right)=\frac{M_{0}\;-\;M_{1}}{M_{0}}\times 100$$

where *M*_0_ is the mass of the emulsified sausage before cooking, and *M*_1_ is the mass of the emulsified sausage after cooking.

### 2.8. Texture Profile Analysis (TPA) of Emulsified Sausages

TPA was performed to evaluate the textural properties of emulsified sausages. Measurements were conducted at the initial stage (0 h) after sample preparation. Samples were equilibrated to room temperature and cut into cubes (1 cm^3^). TPA was carried out using a texture analyzer (TA-XT Plus, Stable Micro Systems, Godalming, Surrey, UK) equipped with a P/36R cylindrical probe (36 mm diameter). TPA was performed using a double compression cycle with a trigger force of 0.5 N, a compression ratio of 60%, and a test speed of 1 mm/s. The recovery time between the two compression cycles was set at 5 s. Hardness, springiness, cohesiveness, and chewiness were selected as the primary TPA parameters to evaluate the textural characteristics of emulsified sausages. The springiness and chewiness values were directly obtained from the instrument software, representing recovery distance (mm) and deformation energy (mJ), respectively.

### 2.9. Determination of TBARS Values in Emulsified Sausages During Refrigerated Storage

Vacuum packaged emulsified sausages were stored at 4 °C for 312 h, and TBARS values were determined at 24, 72, 120, 168, 216, 264, and 312 h. The TBARS assay was used to evaluate the accumulation of secondary lipid oxidation products in the formulated sausage systems. Since different formulations contained different levels of added fat, the TBARS results were interpreted together with the formulation differences rather than as a direct comparison of antioxidant capacity alone. TBARS values were measured according to the method of Summo et al. [21], with slight modifications. Briefly, sausage samples (5.0 g) were placed in test tubes, mixed with 1.5 mL of TBA solution and 8.5 mL of TCA solution, and thoroughly homogenized. The mixture was heated in a water bath at 95 °C for 30 min and then rapidly cooled in an ice-water bath. After cooling, 5 mL of the supernatant was collected and mixed with an equal volume of chloroform, followed by centrifugation at 4500× *g* for 10 min. The absorbance of the upper phase was measured at 532 nm, and the TBARS value was calculated according to the following equation.

(3) $$TBARS\;(mg\;MDA/kg)=\left(\frac{ABS}{W}\right)\times 9.48$$

where ABS represents the absorbance value of the sample at 532 nm; *W* is the sample weight (g).

### 2.10. Sensory Quality Assessment of Emulsified Sausage

Sensory evaluation of emulsified sausages was conducted at the initial stage (day 0) using a to compare the sensory attribute differences among different formulations before storage [22]. A structured sensory evaluation based on a nine-point scoring system was applied by trained evaluators to assess sensory characteristics, including appearance, flavor, taste, mouthfeel, and texture, among different sausage formulations. The evaluation was performed to characterize formulation-induced variations in sensory attributes rather than to assess consumer liking or acceptance.

A sensory panel (*n* = 10) composed of male and female assessors with experience in food science and formal sensory training was recruited. Panelists were trained to identify and score sensory attributes including appearance, texture, flavor, juiciness, and mouthfeel characteristics according to predefined evaluation criteria. Panel members underwent preliminary screening and standardized training sessions to ensure familiarity with sensory attributes and the scoring method, and to enhance consistency and repeatability in evaluations, as recommended in sensory analysis guidelines. Evaluators were instructed to independently assess appearance, texture, flavor, juiciness, and mouthfeel characteristics using visual, olfactory, gustatory, and tactile senses. A double-masked protocol was applied to avoid bias, and panelists scored samples using the provided evaluation forms without discussion. Between sample groups, participants rinsed with room-temperature water to minimize carryover effects. The specific sensory scoring criteria are shown in Table 2.

### 2.11. Statistical Analysis

All experiments were performed in three independent replicates, and results are presented as mean ± standard deviation. For comparison among all sausage formulations, one-way analysis of variance (ANOVA) was conducted using SPSS (version 22.0, IBM Corp., Armonk, NY, USA), and Tukey’s multiple comparison test was applied for comparisons among three or more groups (*p* < 0.05). In addition, two-way ANOVA was performed for the plant oil pre-emulsion treatment groups to evaluate the effects of fat replacement level (50% and 100%), stabilizing system (MP, MP-AG, and MP-GA-AG), and their interaction. The detailed results of the two-way ANOVA are provided in Supplementary Table S1. Figures were generated using Origin (version 9.0, OriginLab Corporation, Northampton, MA, USA).

## 3. Results

### 3.1. Effect of Different Formulations on the Color and pH Value of Emulsified Sausage

**Figure 1:**
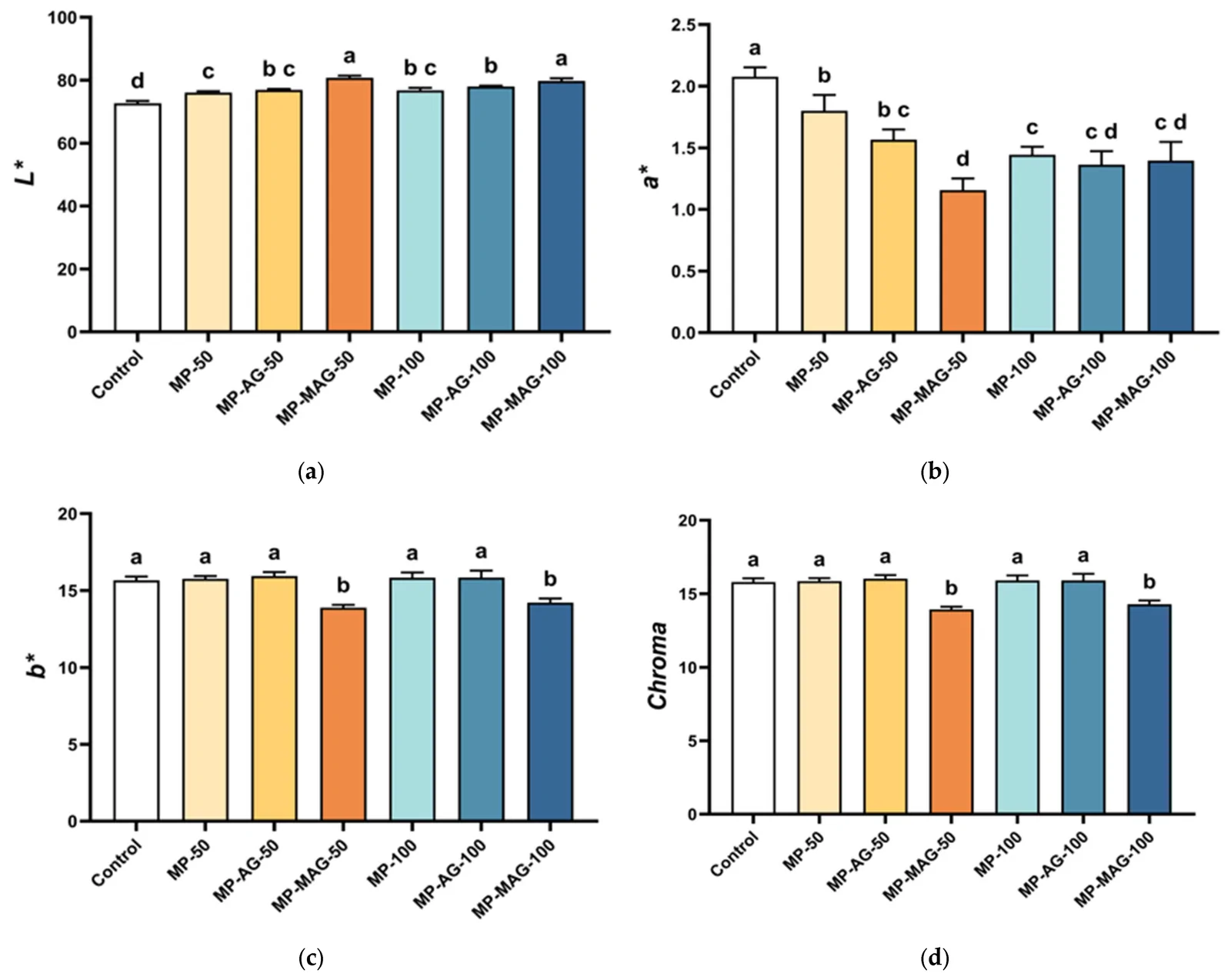
The color of emulsified sausages prepared from different recipes. After replacing the animal fat in the emulsified sausage formula, (**a**) represents brightness, (**b**) indicates redness, (**c**) represents yellowness, (**d**) represents color saturation, and lowercase letters (a–d) indicate significant differences between different treatment groups (*p* < 0.05). The abscissa represents the animal fat control group, 50% vegetable oil pre-emulsification, and 100% vegetable oil pre-emulsification, respectively.

Color and pH are important physicochemical indicators of emulsified sausages, particularly when pork back fat is partially or completely replaced with plant oil pre-emulsions, because visible color changes can directly affect consumer perception and product acceptance. In contrast, pH variations may reflect changes in the chemical environment and overall stability of the meat matrix. As shown in Figure 1, replacement of pork back fat with soybean oil-based pre-emulsions generally resulted in lighter and less red emulsified sausages compared with the animal fat control. Compared with the animal fat control, partial (50%) and complete (100%) replacement of pork back fat with plant oil pre-emulsion significantly increased the L* value and decreased the a* value of the emulsified sausages (*p* < 0.05). In contrast, the b* value and chroma showed no significant change (*p* > 0.05). 

These results indicate that reformulation with soybean oil-based pre-emulsions altered the visual appearance of emulsified sausages, which may lead to a paler color and a reduced resemblance to conventional pork-fat emulsified sausages. Similar effects of plant oil incorporation on the color characteristics of reformulated meat products have also been reported by Youssef and Barbut [23].

The increase in L* value following the incorporation of soybean oil pre-emulsions may be related to changes in lipid dispersion and light scattering within the meat matrix. Compared with solid pork back fat, the pre-emulsified soybean oil droplets stabilized by MP may have promoted a more uniform lipid dispersion, thereby affecting light reflection and contributing to the increased brightness of the sausages [24]. The decrease in a* value may be partly attributed to the lighter color of soybean oil and its lower contribution to redness relative to pork back fat. In addition, differences in lipid distribution and the optical environment of the meat matrix may also influence the visual expression of redness [25].

The effects of different stabilizer systems on color parameters were relatively limited. The incorporation of MP-AG pre-emulsion did not significantly alter a*, b*, or chroma values compared with the MP system (*p* > 0.05). In contrast, the MP-GA-AG pre-emulsion resulted in lower a* and b* values (*p* < 0.05), suggesting that the GA-AG-containing pre-emulsion system may further modify the color characteristics of reformulated sausages.

This change may be associated with the introduction of GA-derived phenolic groups, which could influence pigment-related optical properties through interactions within the meat matrix. However, the specific molecular interactions involved require further investigation [26].

**Figure 2:**
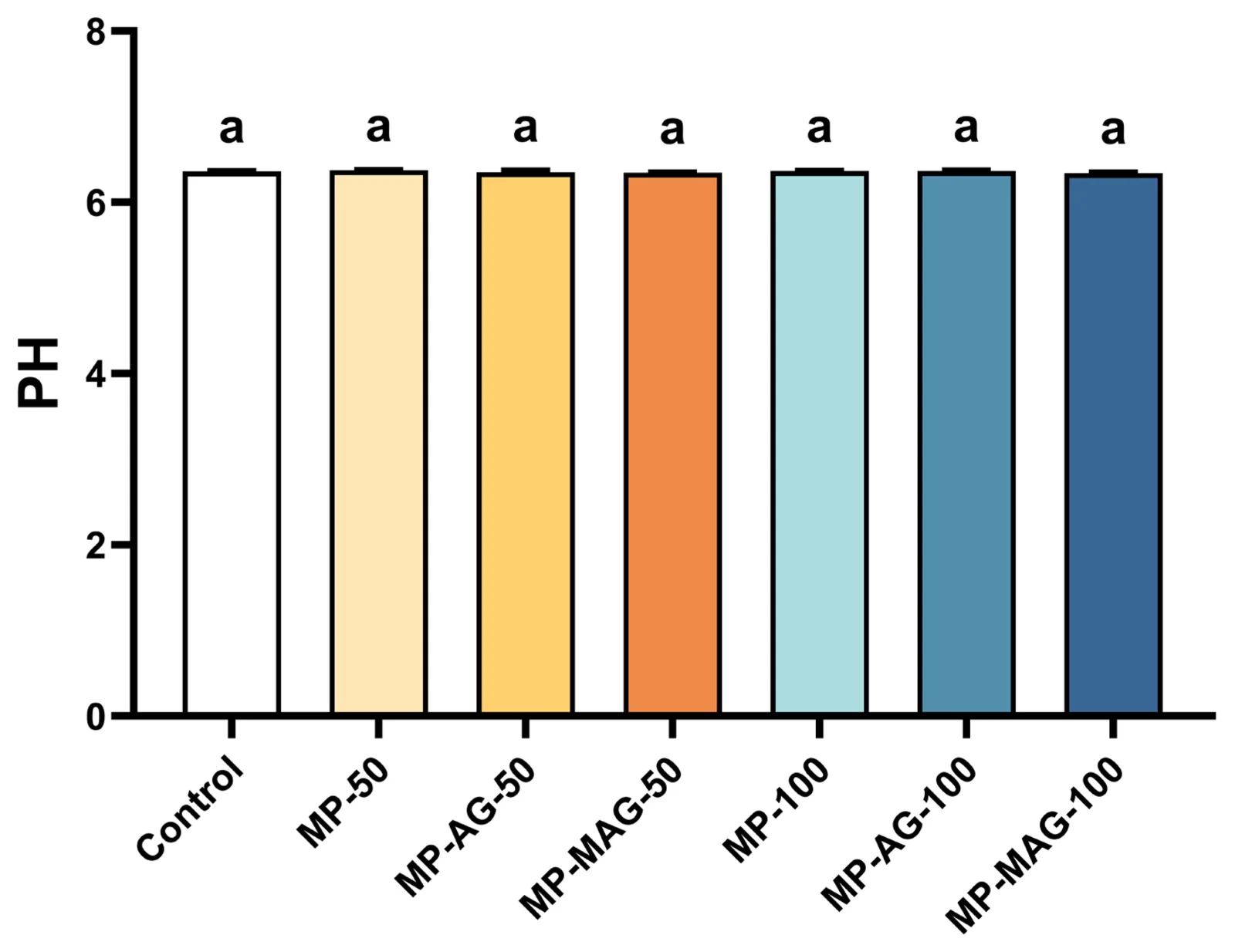
pH of emulsified sausage prepared with different formulations. The abscissa represents the animal fat control group, 50% vegetable oil pre-emulsion, and 100% vegetable oil pre-emulsion, respectively. The same letter are not significantly different (*p* > 0.05).

The pH values of the emulsified sausages are shown in Figure 2. Overall, neither partial nor complete replacement of pork back fat with plant oil pre-emulsion, nor the incorporation of natural or modified AG, significantly affected the pH of the emulsified sausages (*p* > 0.05). The relatively stable pH values indicate that the incorporation of soybean oil-based pre-emulsions did not substantially alter the acid–base environment of the sausage matrix. The minor variation in pH might be associated with the introduction of GA-derived acidic groups during grafting, which could potentially influence the acid–base balance of the system. However, this effect was not sufficient to significantly alter the pH of emulsified sausages, possibly due to the strong buffering capacity of the meat matrix [27].

### 3.2. Effect of Formulation on Cooking Loss and Water–Fat Retention Characteristics of Emulsified Sausages

**Figure 3:**
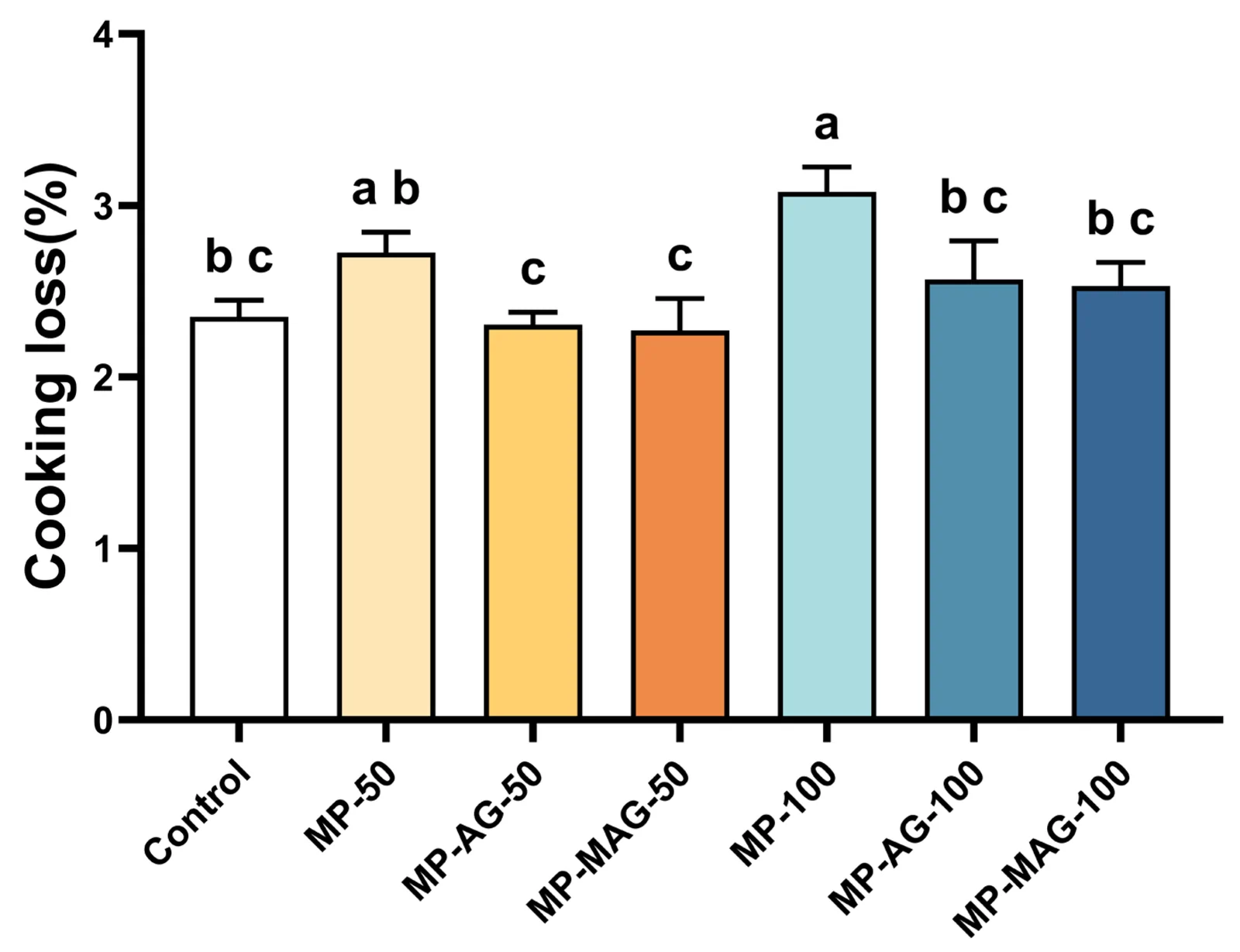
Emulsified sausage cooking losses prepared with different formulations. The lowercase letters (a–c) indicate significant differences between different treatment groups (*p* < 0.05). The abscissa represents the animal fat control group, 50% vegetable oil pre-emulsion, and 100% vegetable oil pre-emulsion, respectively.

Cooking loss is commonly used to evaluate the processing performance of reformulated emulsified sausages because it reflects the ability of the meat matrix to retain water and fat during thermal processing. As shown in Figure 3, replacing pork back fat with the MP-stabilized pre-emulsion significantly reduced the cooking loss of emulsified sausages (*p* < 0.05), suggesting enhanced water and fat retention during thermal processing [28]. A similar trend was reported by Ferro et al. [29], who showed that structured plant oil systems could improve the technological performance of emulsified meat products.

Incorporating GA-AG as an emulsifying stabilizer in the soybean oil-based pre-emulsion further reduced the cooking loss significantly (*p* < 0.05). Compared with the animal fat control, the cooking loss of sausages prepared with the natural AG composite pre-emulsion decreased by 28.75% and 40.37% at the 50% and 100% fat replacement levels, respectively. This improvement may be associated with enhanced emulsion stability and water retention. During pre-emulsification, soybean oil was dispersed into the aqueous phase and coated by MP. At the same time, AG may further contribute to droplet stabilization and matrix reinforcement through its hydrophilic characteristics and possible interactions with protein molecules [30]. During thermal processing, these dispersed oil droplets could be better retained within the heat-induced protein network, thereby limiting the migration and release of water and fat [31]. The water-binding capacity of AG may also promote the formation of a more stable protein-polysaccharide matrix, which is beneficial for reducing cooking loss and improving processing yield. A similar finding was reported by Kim et al. [32], who found that replacing pork back fat with a pre-emulsified grape seed oil system containing gelatin and alginate reduced the cooking loss of emulsified sausages, mainly owing to the water retention of polysaccharides and their interactions with proteins.

However, GA-AG did not further reduce the cooking loss compared with natural AG (*p* > 0.05), although clear differences were still observed among the fat replacement levels. This result suggests that GA grafting may preserve the hydrophilic characteristics and emulsion-stabilizing ability of AG under the present formulation conditions, and that the reduction in cooking loss was governed mainly by the incorporation level of the composite pre-emulsion and the integrity of the meat matrix rather than by the phenolic modification itself [33]. Overall, the reduced cooking loss of the reformulated emulsified sausages may be attributed to the combined effects of pre-emulsified oil incorporation, polysaccharide-assisted stabilization, and improved water–fat retention capacity.

### 3.3. Effect of Different Formulations on the Texture of Emulsified Sausages

Texture is a key quality attribute affecting the consumer acceptance of emulsified sausages, as it reflects the perception of bite, chewiness, and mouthfeel of the final product [34]. As shown in Table 3, replacing pork back fat with the MP-stabilized pre-emulsion altered the textural properties of the emulsified sausages. At the 50% fat replacement level, the MP-stabilized pre-emulsion significantly increased the hardness and springiness of the emulsified sausages compared with the animal fat control (*p* < 0.05). At the same time, chewiness and cohesiveness showed an increasing trend. These changes may be associated with the combined effects of lipid composition, aqueous phase proportion, and the functional properties of the pre-emulsion system, suggesting that the improved texture resulted from multiple formulation-related factors. The enhanced texture could be partly related to the improved dispersion state of soybean oil after pre-emulsification. With MP stabilization, soybean oil dispersed as fine droplets within the aqueous phase, which may facilitate a more uniform distribution of the lipid phase within the meat matrix. Similar findings have been reported in emulsion-based fat replacement systems, in which stable meat emulsions with evenly distributed oil droplets contributed to lower cooking loss by retaining water and fat within the gel matrix [35]. However, the contribution of other formulation-related factors, including differences in protein and polysaccharide incorporation and changes in the physical state of lipid components, should also be considered when interpreting these textural variations.

Notably, when the fat replacement level was increased to 100%, the textural parameters of the sausages prepared with the MP-stabilized pre-emulsion tended to decrease relative to those at the 50% level, although the differences were not significant. This slight decrease might be associated with the increased proportion of soybean oil-based pre-emulsion and the corresponding changes in the composition and physical characteristics of the fat phase. Nevertheless, the non-significant decrease indicated that MP-stabilized pre-emulsification partially compensated for the loss of solid fat functionality and maintained the basic structural integrity of the emulsified sausages [36].

When AG was further incorporated into the MP-stabilized pre-emulsion as a stabilizer, the textural properties of the emulsified sausages were improved to varying degrees compared with those prepared with the MP-stabilized pre-emulsion alone. In particular, at the 50% fat replacement level, the MP-AG system showed significantly higher hardness and springiness than the MP-only system (*p* < 0.05). This result was consistent with the reduction in cooking loss and agreed with previous reports on emulsion-based fat substitutes in sausages [37]. This improvement may be related to the additional stabilization effect provided by AG, which could influence the water retention ability and structural organization of the protein–polysaccharide matrix. Through its hydrophilic properties and potential interactions with protein components, AG may help maintain the distribution of water and oil droplets during thermal processing [38].

Furthermore, no significant differences in textural properties were observed between sausages prepared with the MP-GA-AG composite pre-emulsion and those prepared with the MP-AG system (*p* > 0.05). This result suggests that GA grafting did not further enhance the texture-forming performance of the pre-emulsion system under the current formulation conditions. Previous studies have reported that phenolic modification of polysaccharides may alter their functional properties while maintaining their ability to participate in interfacial stabilization [39]. Therefore, the similar textural performance between AG- and GA-AG-containing systems may indicate that the main contribution of GA-AG was related to functional improvement of the pre-emulsion rather than additional reinforcement of the sausage gel structure.

Overall, these results suggest that the combined effects of lipid replacement strategy, pre-emulsion structure, and the functional roles of protein and polysaccharide components governed the texture changes in reformulated emulsified sausages. The MP-AG and MP-GA-AG composite pre-emulsions may help maintain desirable textural characteristics during fat replacement, whereas GA grafting alone did not further improve the textural properties of the final products [40]. In contrast, GA grafting alone did not further improve the textural properties of the final products [40].

### 3.4. Analysis of Sensory Differences in Emulsified Sausages Prepared with Different Formulations

**Figure 4:**
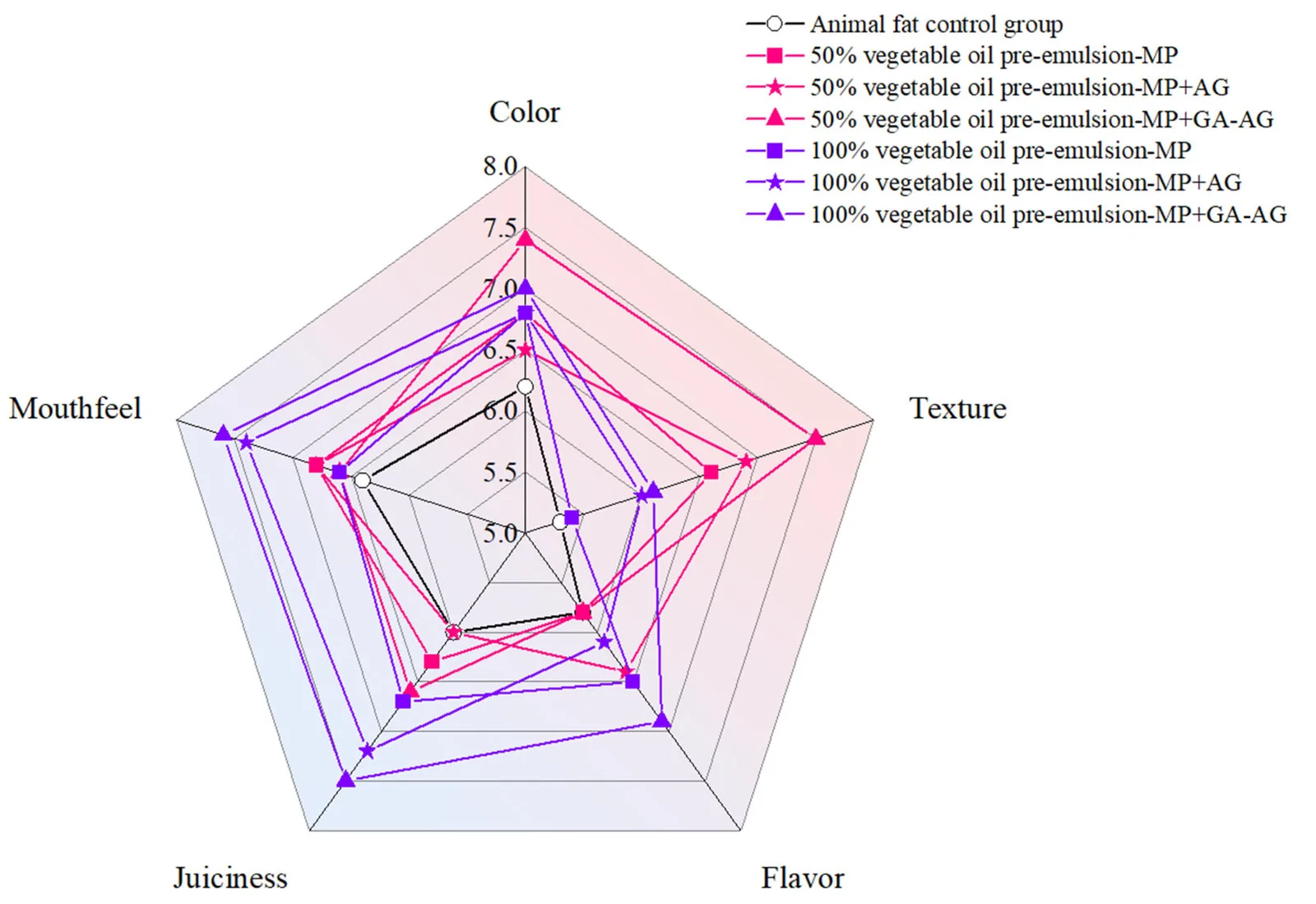
Sensory scores of emulsified sausages prepared with different formulations.

To further assess the application potential of the fat-replaced emulsified sausages, sensory evaluation was performed at the initial stage to determine the sensory characteristics of the different formulations. As shown in Figure 4, the sausages prepared with plant oil pre-emulsions exhibited different sensory profiles compared with the pork back fat control. 

The detailed sensory scores, standard deviations, and statistical comparisons among formulations are presented in Table 4. 

Considering the results described above, these differences may be associated with variations in textural properties among formulations (Table 3), which could influence the sensory perception of emulsified sausages.

When pork back fat was partially replaced with the plant oil pre-emulsion, the sensory scores of the emulsified sausages were generally improved, with more pronounced increases in the color and texture scores at the 50% replacement level. This observation was consistent with the instrumental texture results, suggesting that incorporating a pre-emulsion system may influence the perceived structural characteristics of the products [41]. The incorporation of AG further affected the sensory profile, particularly by improving the texture score, which may be related to the functional contribution of AG within the MP-based pre-emulsion system. Rather than directly acting as a fat replacer, AG served as a stabilizing component of the pre-emulsion and may influence the sensory properties by modifying the characteristics of the sausage matrix. However, the juiciness score decreased after AG incorporation at the 50% replacement level, probably because the increased firmness of the AG-containing sausages may have affected the perception of juiciness during mastication, despite their relatively lower cooking loss values [42].

At the 100% replacement level, the emulsified sausages received higher taste, juiciness, and flavor scores, which could be associated with the altered lipid composition and the incorporation of soybean oil-based pre-emulsion into the sausage formulation. Plant oil has greater fluidity than solid pork back fat, and the different physical state of the lipid phase may contribute to differences in mouthfeel perception during oral processing [43]. In addition, the reduced cooking loss observed in the complete replacement groups suggested improved retention of moisture and lipid components during thermal processing, which may partially explain the changes in juiciness perception. After AG incorporation, the taste and juiciness scores were further improved, whereas the flavor score showed a slight decrease. This difference may be related to changes in the composition and structure of the protein–polysaccharide-containing pre-emulsion system, although further investigation is required to clarify the underlying mechanism [33].

The further incorporation of GA-AG maintained or improved the overall sensory acceptability of the emulsified sausages, particularly at the complete replacement level. This result suggests that the addition of GA-AG within the soybean oil-based pre-emulsion system did not negatively affect sensory performance under the present formulation conditions. The reduction in the instrumental a* and b* values observed in the GA-modified AG groups was not accompanied by a decline in the sensory color score, indicating that the measured instrumental color differences did not necessarily translate into reduced sensory evaluation scores. Overall, the sensory results suggest that the performance of GA-AG-containing formulations was mainly related to their role as a functional stabilizing component of the MP-based pre-emulsion rather than as a direct fat replacement ingredient.

### 3.5. Effect of Different Formulations on TBARS Values of Emulsified Sausages

**Figure 5:**
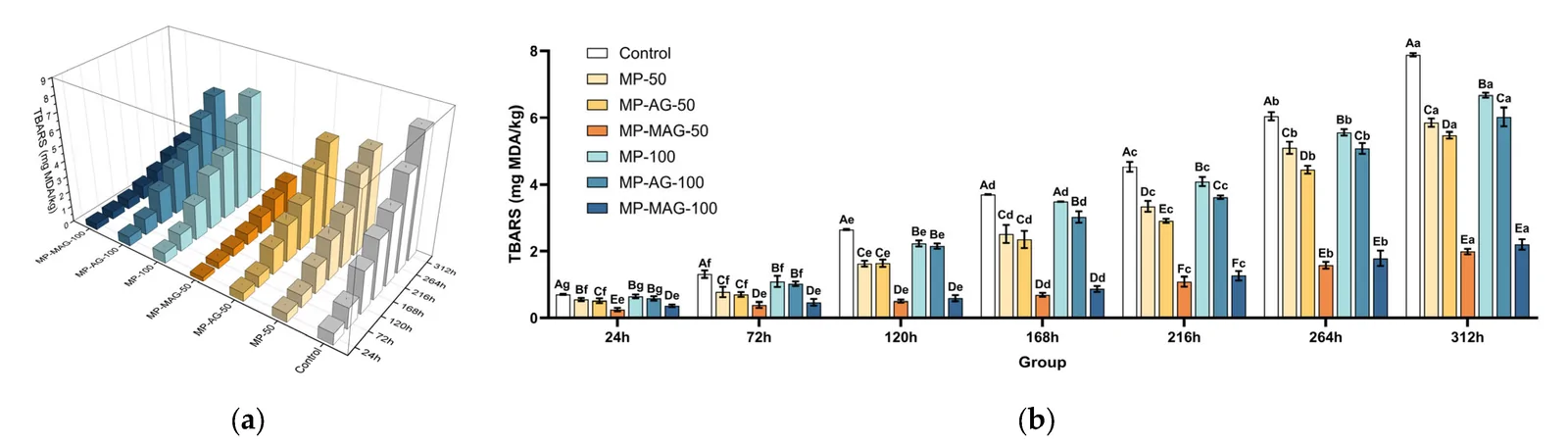
Changes in TBARS values of emulsified sausages prepared with different formulations during refrigerated storage. (**a**) TBARS trend chart and (**b**) significant trend. Uppercase letters (A–F) indicate significant differences between different treatment groups at the same measurement time (*p* < 0.05). The lowercase letters (a–g) indicate that there was a significant difference between the same treatment group at different measurement times (*p* < 0.05). Among them, 50% on the horizontal axis represents a 50% plant oil pre-emulsion, and 100% represents a 100% plant oil pre-emulsion.

Lipid oxidation is an important factor affecting the quality of meat products; therefore, TBARS values were determined to evaluate the accumulation of secondary lipid oxidation products in emulsified sausages during refrigerated storage. In this study, TBARS values were used as an indicator of malondialdehyde (MDA) formation, a major secondary product generated during lipid oxidation. As shown in Figure 5 and Table 5, the TBARS values increased progressively in all formulations during storage. In contrast, the sausages prepared with pork back fat exhibited the highest degree of lipid oxidation throughout the storage period. This result indicates that the pork back fat formulation showed greater accumulation of lipid oxidation products under the investigated storage conditions.

However, the reformulated sausages contained different lipid compositions and lower amounts of added animal fat than the control group. Therefore, the lower TBARS values observed in the reformulated products should not be interpreted solely as an antioxidant effect, because the reduced lipid content may decrease the amount of available substrates for oxidation. The differences among formulations may reflect the combined influence of lipid composition, substrate availability, and the interfacial functionality of the pre-emulsion system.

Replacing 50% of the pork back fat with the plant oil pre-emulsion significantly reduced TBARS accumulation (*p* < 0.05), suggesting that the incorporation of the soybean oil-based pre-emulsion affected the oxidation behavior of the sausage matrix. This effect may be attributed to the adsorption of MP at the surface of the oil droplets, forming an interfacial layer that could reduce the exposure of the lipid phase to oxygen and other pro-oxidants. Similar effects have been reported for protein-stabilized plant oil emulsions, where the interfacial protein layer contributed to improved physical protection of dispersed lipid droplets [34,44]. The incorporation of AG slightly delayed lipid oxidation, probably because AG improved the physical stability of the composite pre-emulsion by increasing the viscosity of the aqueous phase and providing steric hindrance. Nevertheless, the contribution of native AG to oxidative protection may be limited because its primary role is associated with emulsion stabilization rather than direct antioxidant activity.

Compared with native AG, GA-AG showed a markedly stronger inhibitory effect on lipid oxidation. The GA-AG-treated sausages exhibited the lowest TBARS values throughout storage, and at day 13 the TBARS value was only 32.8% of that in the MP-treated group. This reduction may be explained by the combined effects of improved interfacial protection and the antioxidant potential introduced by the grafted phenolic groups. GA-AG could cooperate with MP to form a more protective interfacial layer around the oil droplets, thereby reducing the contact between the lipid phase and pro-oxidants. Moreover, phenolic compounds derived from gallic acid modification have been reported to possess radical-scavenging and metal-chelating abilities, which may contribute to the reduced formation of secondary lipid oxidation products [14]. However, further molecular investigations are required to confirm the specific interactions involved in this system.

At the 100% fat replacement level, the TBARS values showed a trend similar to that observed at the 50% level. However, the AG and GA-AG-treated groups exhibited higher TBARS values than the corresponding groups at the 50% level, which may be associated with the increased amount of soybean oil incorporated through the pre-emulsion system and the corresponding increase in available lipid substrates for oxidation [45]. Overall, the GA-AG-containing soybean oil pre-emulsion system showed potential for regulating lipid oxidation behavior in fat-reduced emulsified sausages under the present formulation conditions. Nevertheless, the observed TBARS differences should be interpreted considering both the compositional changes caused by fat replacement and the functional contribution of the pre-emulsion system.

## 4. Conclusions

This study showed that soybean oil-based pre-emulsions stabilized by MP, MP-AG, or MP-GA-AG can serve as pork back fat substitutes in Western-style emulsified sausages, and clarified the practical role of GA-AG as an emulsifying stabilizer and antioxidant component within the pre-emulsion system in a real meat matrix. Partial (50%) and complete (100%) replacement of pork back fat improved the initial processing performance of the sausages, as reflected by the reduced cooking loss and enhanced textural properties. The incorporation of AG further reduced cooking loss, whereas GA-AG maintained the physical stabilizing function of AG without compromising sensory acceptability. Among the tested formulations, the 50% fat replacement level provided a favorable balance between structural improvement and sensory quality, indicating its potential applicability for reduced-animal-fat sausage formulation.

These findings indicate the feasibility of using GA-AG in MP-stabilized soybean oil pre-emulsions to combine interfacial stabilization and antioxidant protection in MP-stabilized plant oil pre-emulsions for reduced-animal-fat emulsified sausages. During refrigerated storage, the GA-AG-treated sausages showed the strongest inhibition of TBARS accumulation, with the TBARS value at day 13 reduced to 32.8% of that in the MP-only system. Considering the differences in lipid composition among formulations, this reduction in TBARS values may reflect the combined effects of formulation composition and the functional contribution of the GA-AG-stabilized pre-emulsion system. Overall, the MP-GA-AG-stabilized soybean oil pre-emulsion system provides a promising strategy for improving the processing performance and regulating lipid oxidation behavior in reduced-animal-fat emulsified sausages.

## Figures and Tables

**Table 1:** Basic formulation of emulsified sausages (g/batch and percentage of final formulation).

Basic Formula g (%)	Processing Group						
	Animal Fat	50% Vegetable Oil			100% Vegetable Oil		
	Control Group	MP	MP + AG	MP + MAG	MP	MP + AG	MP +MAG
Porcine longissimus dorsi muscle	120 (60)	120 (60)	120 (60)	120 (60)	120 (60)	120 (60)	120 (60)
Pig back fat	30 (15)	15 (7.5)	15 (7.5)	15 (7.5)	-	-	-
Ice	45.4 (22.7)	30 (15)	30 (15)	30 (15)	15 (7.5)	15 (7.5)	15 (7.5)
Pre-emulsion	-	30.4 (15.2)	30.4 (15.2)	30.4 (15.2)	60.4 (30.2)	60.4 (30.2)	60.4 (30.2)
Salt	4 (2)	4 (2)	4 (2)	4 (2)	4 (2)	4 (2)	4 (2)
Tripolyphosphate	0.6 (0.3)	0.6 (0.3)	0.6 (0.3)	0.6 (0.3)	0.6 (0.3)	0.6 (0.3)	0.6 (0.3)
Total weight	200 (100)	200 (100)	200 (100)	200 (100)	200 (100)	200 (100)	200 (100)

Note: Replacement levels were defined according to the reduction in pork back fat relative to the 30 g used in CK and did not represent equivalent lipid replacement. The pre-emulsions contained 20% (*w*/*w*) soybean oil based on their total mass (oil-to-aqueous phase mass ratio, 20:80); thus, 30.4 and 60.4 g of pre-emulsion supplied 6.08 and 12.08 g of soybean oil, respectively.

**Table 2:** Sensory evaluation criteria.

Project	Evaluation Criteria	Score
Color	The shape is complete, the color of the facet is normal, and there is no bad color such as whitishness	7–9
	The shape is more complete, and the color of the section is more normal, with a slight whitish or dark red	4–6
	The shape is incomplete, the facet is broken, and the color is whitish or crimson	1–3
Texture	The cutting surface is fine and uniform, and the small holes are evenly distributed and dense	7–9
	The slice surface is fine and uniform, and the aperture is slightly larger but still evenly distributed	4–6
	The slice surface is rough, the pore size is large and unevenly distributed	1–3
Flavor	It has a meaty flavor and no peculiar smell, such as sweetness, bitterness, astringency, etc.	7–9
	It has a meaty flavor but has a slightly bitter, sweet, or astringent taste	4–6
	The meat has a small flavor and has a bitter, sweet, or astringent taste	1–3
Juiciness	High juiciness with abundant moisture release during chewing	7–9
	Moderate juiciness with acceptable moisture release	4–6
	Low juiciness with a dry mouthfeel	1–3
mouthfeel	Tender, smooth, and cohesive texture with good chewability; no noticeable graininess or dryness.	7–9
	Moderately tender and chewable texture with slight graininess or reduced cohesiveness.	4–6
	Firm, coarse, or poorly cohesive texture with obvious dryness and unpleasant mouthfeel	1–3

**Table 3:** Texture characteristics of emulsified sausages prepared by different formulations.

Processing Groups	Hardness/N	Springiness/mm	Chewiness/mj	Cohesiveness
CK	15.97 ± 0.85 ^c^	3.12 ± 0.12 ^c^	26.95 ± 2.38 ^b^	0.37 ± 0.09 ^c^
MP-50	18.13 ± 0.40 ^b^	3.69 ± 0.21 ^b^	29.45 ± 2.57 ^ab^	0.39 ± 0.03 ^bc^
MP-AG-50	20.23 ± 0.45 ^a^	3.92 ± 0.25 ^a^	31.43 ± 2.08 ^ab^	0.47 ± 0.03 ^ab^
MP-MAG-50	20.10 ± 0.72 ^a^	3.90 ± 0.30 ^a^	32.78 ± 1.96 ^a^	0.49 ± 0.04 ^a^
MP-100	16.97 ± 0.42 ^bc^	3.51 ± 0.21 ^b^	28.09 ± 3.16 ^ab^	0.35 ± 0.01 ^c^
MP-AG-100	18.00 ± 0.26 ^b^	3.79 ± 0.24 ^b^	30.86 ± 1.32 ^ab^	0.42 ± 0.04 ^ab^
MP-MAG-100	18.27 ± 1.02 ^b^	3.87 ± 0.16 ^a^	30.87 ± 0.96 ^ab^	0.41 ± 0.02 ^bc^

Note: The lowercase letters (a–c) indicate significant differences between different treatment groups (*p* < 0.05).

**Table 4:** Sensory evaluation scores and statistical analysis of emulsified sausages with different formulations.

Processing Groups	Color	Texture	Flavor	Juiciness	Mouthfeel
CK	6.20 ± 0.43 ^d^	5.30 ± 0.54 ^d^	5.80 ± 0.46 ^c^	6.00 ± 0.61 ^c^	6.40 ± 0.50 ^b^
MP-50	6.80 ± 0.34 ^bc^	6.60 ± 0.39 ^b^	5.80 ± 0.44 ^c^	6.30 ± 0.27 ^bc^	6.80 ± 0.49 ^b^
MP-AG-50	6.50 ± 0.48 ^cd^	6.90 ± 0.40 ^b^	6.40 ± 0.32 ^b^	6.00 ± 0.50 ^c^	6.80 ± 0.52 ^b^
MP-MAG-50	7.40 ± 0.32 ^a^	7.50 ± 0.24 ^a^	5.80 ± 0.44 ^c^	6.60 ± 0.36 ^b^	6.60 ± 0.74 ^b^
MP-100	6.80 ± 0.3 ^bc^	5.40 ± 0.45 ^d^	6.50 ± 0.28 ^ab^	6.70 ± 0.45 ^b^	6.60 ± 0.32 ^b^
MP-AG-100	6.80 ± 0.60 ^bc^	6.00 ± 0.42 ^c^	6.10 ± 0.37 ^bc^	7.20 ± 0.38 ^a^	7.40 ± 0.45 ^a^
MP-MAG-100	7.00 ± 0.38 ^ab^	6.10 ± 0.51 ^c^	6.90 ± 0.37 ^a^	7.50 ± 0.28 ^a^	7.60 ± 0.42 ^a^

Note: Values are expressed as mean ± standard deviation (*n* = 10). Different lowercase letters within the same column indicate significant differences among treatments (*p* < 0.05).

**Table 5:** TBARS values of emulsified sausages prepared with different formulations during refrigerated storage.

Groups	Storage Time						
	24 h	72 h	120 h	168 h	216 h	264 h	312 h
Control	0.71 ± 0.02 ^Ag^	1.31 ± 0.11 ^Af^	2.65 ± 0.01 ^Ae^	3.70 ± 0.01 ^Ad^	4.53 ± 0.15 ^Ac^	6.05 ± 0.12 ^Ab^	7.88 ± 0.05 ^Aa^
MP-50	0.56 ± 0.04 ^Bf^	0.78 ± 0.16 ^Cf^	1.63 ± 0.09 ^Ce^	2.52 ± 0.27 ^Cd^	3.35 ± 0.16 ^Dc^	5.1 ± 0.18 ^Cb^	5.86 ± 0.13 ^Ca^
MP-AG-50	0.52 ± 0.07 ^Cf^	0.71 ± 0.07 ^Cf^	1.64 ± 0.11 ^Ce^	2.35 ± 0.26 ^Cd^	2.91 ± 0.06 ^Ec^	4.44 ± 0.12 ^Db^	5.48 ± 0.10 ^Da^
MP-MAG-50	0.25 ± 0.05 ^Ee^	0.4 ± 0.09 ^De^	0.51 ± 0.04 ^De^	0.69 ± 0.06 ^Dd^	1.09 ± 0.15 ^Fc^	1.58 ± 0.10 ^Eb^	1.99 ± 0.08 ^Ea^
MP-100	0.65 ± 0.06 ^Bg^	1.09 ± 0.16 ^Bf^	2.23 ± 0.09 ^Be^	3.48 ± 0.01 ^Ad^	4.09 ± 0.13 ^Bc^	5.56 ± 0.09 ^Bb^	6.68 ± 0.08 ^Ba^
MP-AG-100	0.59 ± 0.06 ^Bg^	1.03 ± 0.07 ^Bf^	2.15 ± 0.08 ^Be^	3.03 ± 0.17 ^Bd^	3.62 ± 0.05 ^Cc^	5.08 ± 0.16 ^Cb^	6.02 ± 0.28 ^Ca^
MP-MAG-100	0.37 ± 0.04 ^De^	0.47 ± 0.10 ^De^	0.59 ± 0.09 ^De^	0.87 ± 0.09 ^Dd^	1.27 ± 0.14 ^Fc^	1.79 ± 0.23 ^Eb^	2.20 ± 0.16 ^Ea^

Note: Uppercase letters (A–F) indicate significant differences between different treatment groups at the same measurement time (*p* < 0.05). The lowercase letters (a–g) indicate that there was a significant difference between the same treatment group at different measurement times (*p* < 0.05).

## Data Availability

The original contributions presented in this study are included in the article/Supplementary Materials. Further inquiries can be directed to the corresponding authors.

## Supplementary Materials

The following supporting information can be downloaded at: https://www.mdpi.com/article/10.3390/foods15162795/s1. Table S1: Effects of fat replacement level and stabilizing system on quality characteristics of emulsified sausages analyzed by two-way ANOVA.

## Abbreviations

The following abbreviations are used in this manuscript:

MP	Myofibrillar protein
AG	Arabic gum
GA	Gallic acid
GA-AG	GA-modified AG
MP-MAG	Myofibrillar protein-modified AG
