# Supplementary material for: Effects of Myofibrillar Protein-Stabilized Soybean Oil Pre-Emulsions Containing Arabic Gum or Gallic Acid-Grafted Arabic Gum on the Quality Characteristics and Oxidative Stability of Reduced-Animal-Fat Emulsified Sausages
Source: Foods. 2026 Aug 10;15(16):2795. doi: 10.3390/foods15162795 (PMC13511775; doi:10.3390/foods15162795)
Supplement: Supplementary file 1 [file foods-15-02795-s001.zip › foods-4463690-supplementary.pdf]

Table S1. Effects of fat replacement level and stabilizing system on quality characteristics of emulsified sausages analyzed by two-way ANOVA.

Response variable	*P* value		
	Fat replacement level	Stabilizing system	Fat replacement level × stabilizing system
PH	0.824	0.151	0.677
*L**	0.505	< 0.001	0.056
a*	0.117	0.003	0.006
b*	0.562	< 0.001	0.599
C*	0.608	< 0.001	0.542
Cooking loss	0.002	< 0.001	0.837
Hardness	< 0.001	0.008	0.545
Elasticity	0.326	0.104	0.869
Chewiness	0.228	0.068	0.864
Cohesiveness	0.004	0.001	0.589
Color	0.707	< 0.001	0.007
Texture	< 0.001	< 0.001	0.064
Flavor	< 0.001	0.123	< 0.001
Juiciness	< 0.001	< 0.001	< 0.001
Palate	< 0.001	0.003	< 0.001

Note: Two-way ANOVA was performed only for plant oil pre-emulsion replacement groups (excluding CK).
